# 2-{[3-Methyl-4-(2,2,2-trifluoro­eth­oxy)pyridin-2-yl]methyl­sulfan­yl}-1*H*-benzimidazole propan-2-ol monosolvate: a second monoclinic polymorph

**DOI:** 10.1107/S1600536812022143

**Published:** 2012-06-13

**Authors:** Jin-Ju Ma, Ming-Hui Qi, Ming-Huang Hong, Jie Lu, Guo-Bin Ren

**Affiliations:** a School of Chemical Engineering and Energy, Zhengzhou University, Zhengzhou 450001, People’s Republic of China; b Pharmaceutical Crystal Engineering Research Group, Shanghai Institute of Pharmaceutical Industry, 1320 Beijing Road (West), Shanghai 200040, People’s Republic of China; c National Engineering Laboratory for Cereal Fermentation Technology, School of Chemical & Material Engineering, Jiangnan University, Wuxi 214122, People’s Republic of China

## Abstract

In the crystal structure of the title compound, C_16_H_14_F_3_N_3_OS·C_3_H_8_O, the mol­ecules are linked into chains along [010] *via* N—H⋯O and O—H⋯N hydrogen bonds. The triclinic form was reported by Ren *et al.* [(2011). *Acta Cryst*. E**67**, o270] and the first monoclinic form by Chen *et al.* [(2012). *Acta Cryst.* E**68**, o2015–o2016]. The fused five-and six-membered rings make a dihedral angle of 1.22 (2)°, while the benzene and pyridine rings make a dihedral angle of 10.15 (2)°.

## Related literature

For the use of the title compound as an inter­mediate in the synthesis of the anti-ulcer drug lansoprazole {systematic name: (*RS*)-2-([3-methyl-4-(2,2,2-trifluoro­eth­oxy)pyridin-2-yl]methyl­sulfin­yl)-1*H*-benzo[*d*]imidazole}, see: Del Rio *et al.* (2007 ▶); Reddy *et al.* (2008 ▶); Iwahi *et al.* (1991 ▶). For related structures, see: Swamy & Ravikumar (2007 ▶); Hakim *et al.* (2010 ▶). For the triclinic polymorph of the title propan-2-ol solvo-polymorph, see: Ren *et al.* (2011 ▶) and for the monoclinic mono­hydrate, see: Chen *et al.* (2012 ▶).
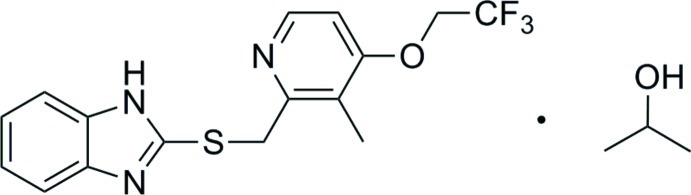


## Experimental

### 

#### Crystal data

C_16_H_14_F_3_N_3_OS·C_3_H_8_O
*M* *_r_* = 413.46
Monoclinic, 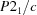

*a* = 17.4583 (2) Å
*b* = 7.4162 (1) Å
*c* = 16.9622 (2) Å
β = 116.255 (2)°
*V* = 1969.60 (5) Å^3^
*Z* = 4
Cu *K*α radiation
μ = 1.89 mm^−1^
*T* = 296 K
0.31 × 0.23 × 0.15 mm

#### Data collection

Bruker APEXII diffractometer
Absorption correction: multi-scan (*SADABS*; Bruker, 2009 ▶) *T*_min_ = 0.592, *T*_max_ = 0.765
13351 measured reflections
3410 independent reflections
3266 reflections with *I* > 2σ(*I*)
*R*_int_ = 0.017

#### Refinement

*R*[*F* ^2^ > 2σ(*F* ^2^)] = 0.040
*wR*(*F* ^2^) = 0.116
*S* = 1.06
3410 reflections
254 parameters
H-atom parameters constrained
Δρ_max_ = 0.38 e Å^−3^
Δρ_min_ = −0.28 e Å^−3^

### 

Data collection: *APEX2* (Bruker, 2009 ▶); cell refinement: *SAINT* (Bruker, 2009 ▶); data reduction: *SAINT*; program(s) used to solve structure: *SHELXS97* (Sheldrick, 2008 ▶); program(s) used to refine structure: *SHELXL97* (Sheldrick, 2008 ▶); molecular graphics: *SHELXTL* (Sheldrick, 2008 ▶); software used to prepare material for publication: *SHELXTL*.

## Supplementary Material

Crystal structure: contains datablock(s) I, global. DOI: 10.1107/S1600536812022143/bg2460sup1.cif

Structure factors: contains datablock(s) I. DOI: 10.1107/S1600536812022143/bg2460Isup2.hkl

Supplementary material file. DOI: 10.1107/S1600536812022143/bg2460Isup3.cml

Additional supplementary materials:  crystallographic information; 3D view; checkCIF report

## Figures and Tables

**Table 1:** Hydrogen-bond geometry (Å, °)

*D*—H⋯*A*	*D*—H	H⋯*A*	*D*⋯*A*	*D*—H⋯*A*
O2—H2*B*⋯N1^i^	0.82	2.01	2.8142 (18)	167
N2—H2*A*⋯O2^ii^	0.86	1.98	2.8027 (18)	161

Symmetry codes: (i) 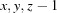; (ii) 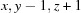.

**Fig. 1.**
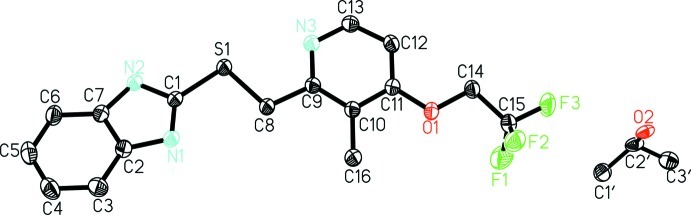
The content of asymmetric unit (I) showing the atom-labelling scheme. Displacement ellipsoids are drawn at the 30% probability level. Dashed lines denote hydrogen bonds. H atoms have been omitted for clarity.

**Fig. 2.**
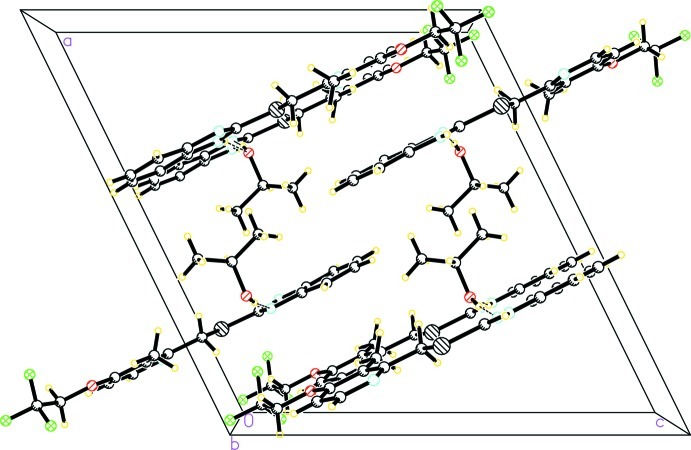
A packing diagram, projected along the [010] chains.

## supplementary crystallographic information

### Comment

Lansoprazole (Del Rio *et al.*, 2007; Reddy *et al.*, 2008) and many of its analogues are characterized by an anti-ulcer effect (Iwahi *et al.*,1991) The title compound, (I), is the critical reaction intermediate of lansoprazole Recently, the compound was successfully crystallized from 2-propanol, and the crystal structure is now firstly reported.

The crystral structure (Fig 1) contains one independent molecule and one 2-propanol solvato which are involved in the formation of hydrogen-bonded chains running along [010] *via* N—H···O and O—H···N hydrogen bonds (Table 1 and Fig. 2). The 2-propanol molecules acts as a hydrogen-bond bridge, providing further stability to the crystal lattice.

### Experimental

The raw material was kindly provided by Shanghai Enran Sci-Tech Investment Management Co., Ltd. The compound was dissolved in 2-propanol and suitable crystals of X-ray were obtained by slow evaporation at room temperature over a period of one week.

### Refinement

All C-H atoms were constrained to an ideal geometry with C—H distances of 0.98 Å and *U*_iso_(H) = 1.2*U*_eq_(C) for CH; 0.97 Å and *U*_iso_(H) = 1.2*U*_eq_ (C) for CH_2_; 0.96 Å and *U*_iso_(H) = 1.5*U*_eq_(C) for CH_3_; 0.82 Å and *U*_iso_(H) = 1.5*U*_eq_(C)for OH atoms; and 0.86 Å and *U*_iso_(H) = 1.5*U*_eq_(C) for NH atoms.

### Crystal data

**Table:**

C_16_H_14_F_3_N_3_OS·C_3_H_8_O	*F*(000) = 864
*M**_r_* = 413.46	*D*_x_ = 1.394 Mg m^−^^3^
Monoclinic, *P*2_1_/*c*	Cu *K*α radiation, λ = 1.54178 Å
Hall symbol: -P 2ybc	Cell parameters from 3266 reflections
*a* = 17.4583 (2) Å	θ = 6.1–67.0°
*b* = 7.4162 (1) Å	µ = 1.89 mm^−^^1^
*c* = 16.9622 (2) Å	*T* = 296 K
β = 116.255 (2)°	Column, colorless
*V* = 1969.60 (5) Å^3^	0.31 × 0.23 × 0.15 mm
*Z* = 4	

### Data collection

**Table:**

Bruker APEXII diffractometer	3410 independent reflections
Radiation source: fine-focus sealed tube	3266 reflections with *I* > 2σ(*I*)
Graphite monochromator	*R*_int_ = 0.017
Detector resolution: 0 pixels mm^-1^	θ_max_ = 67.0°, θ_min_ = 6.1°
phi and ω scans	*h* = −19→20
Absorption correction: multi-scan (*SADABS*; Bruker, 2009)	*k* = −8→8
*T*_min_ = 0.592, *T*_max_ = 0.765	*l* = −20→19
13351 measured reflections	

### Refinement

**Table:**

Refinement on *F*^2^	Secondary atom site location: difference Fourier map
Least-squares matrix: full	Hydrogen site location: inferred from neighbouring sites
*R*[*F*^2^ > 2σ(*F*^2^)] = 0.040	H-atom parameters constrained
*wR*(*F*^2^) = 0.116	*w* = 1/[σ^2^(*F*_o_^2^) + (0.0697*P*)^2^ + 0.5861*P*] where *P* = (*F*_o_^2^ + 2*F*_c_^2^)/3
*S* = 1.06	(Δ/σ)_max_ = 0.002
3410 reflections	Δρ_max_ = 0.38 e Å^−^^3^
254 parameters	Δρ_min_ = −0.28 e Å^−^^3^
0 restraints	Extinction correction: *SHELXL97* (Sheldrick, 2008), Fc^*^=kFc[1+0.001xFc^2^λ^3^/sin(2θ)]^-1/4^
Primary atom site location: structure-invariant direct methods	Extinction coefficient: 0.0024 (3)

### Special details

**Table:**

Geometry. All e.s.d.'s (except the e.s.d. in the dihedral angle between two l.s. planes) are estimated using the full covariance matrix. The cell e.s.d.'s are taken into account individually in the estimation of e.s.d.'s in distances, angles and torsion angles; correlations between e.s.d.'s in cell parameters are only used when they are defined by crystal symmetry. An approximate (isotropic) treatment of cell e.s.d.'s is used for estimating e.s.d.'s involving l.s. planes.
Refinement. Refinement of *F*^2^ against ALL reflections. The weighted *R*-factor *wR* and goodness of fit *S* are based on *F*^2^, conventional *R*-factors *R* are based on *F*, with *F* set to zero for negative *F*^2^. The threshold expression of *F*^2^ > σ(*F*^2^) is used only for calculating *R*-factors(gt) *etc*. and is not relevant to the choice of reflections for refinement. *R*-factors based on *F*^2^ are statistically about twice as large as those based on *F*, and *R*- factors based on ALL data will be even larger.

### Fractional atomic coordinates and isotropic or equivalent isotropic displacement parameters (Å^2^)

**Table:**

	*x*	*y*	*z*	*U*_iso_*/*U*_eq_	
S1	0.22271 (3)	0.11157 (5)	1.05242 (3)	0.04524 (17)	
O1	0.09818 (8)	0.61043 (16)	0.71885 (7)	0.0497 (3)	
O2	0.32047 (8)	0.67645 (16)	0.17242 (10)	0.0573 (4)	
H2B	0.3016	0.5801	0.1800	0.086*	
N1	0.28104 (9)	0.32274 (18)	1.20227 (9)	0.0417 (3)	
N2	0.29844 (8)	0.02492 (18)	1.22301 (8)	0.0419 (3)	
H2A	0.2958	−0.0880	1.2105	0.050*	
N3	0.14436 (10)	0.1600 (2)	0.87641 (9)	0.0476 (4)	
F1	0.13495 (9)	0.8359 (2)	0.61276 (11)	0.0938 (5)	
F2	0.01560 (8)	0.89596 (15)	0.61011 (8)	0.0681 (3)	
F3	0.02040 (11)	0.76215 (19)	0.50116 (8)	0.0893 (5)	
C1	0.26983 (10)	0.1637 (2)	1.16472 (10)	0.0375 (3)	
C1'	0.47122 (13)	0.6930 (4)	0.26857 (17)	0.0747 (6)	
H1'A	0.4639	0.6167	0.3104	0.112*	
H1'B	0.4658	0.8169	0.2816	0.112*	
H1'C	0.5269	0.6734	0.2717	0.112*	
C2	0.32070 (10)	0.2857 (2)	1.29233 (11)	0.0415 (4)	
C2'	0.40380 (12)	0.6487 (3)	0.17709 (15)	0.0594 (5)	
H2'A	0.4095	0.5217	0.1646	0.071*	
C3	0.34706 (13)	0.4014 (3)	1.36387 (13)	0.0556 (5)	
H3B	0.3384	0.5251	1.3561	0.067*	
C3'	0.41146 (15)	0.7633 (4)	0.10808 (17)	0.0729 (6)	
H3'A	0.3673	0.7311	0.0513	0.109*	
H3'B	0.4663	0.7440	0.1092	0.109*	
H3'C	0.4057	0.8880	0.1196	0.109*	
C4	0.38645 (14)	0.3274 (3)	1.44666 (13)	0.0631 (5)	
H4A	0.4048	0.4029	1.4954	0.076*	
C5	0.39947 (13)	0.1429 (3)	1.45925 (13)	0.0595 (5)	
H5A	0.4272	0.0979	1.5162	0.071*	
C6	0.37235 (11)	0.0253 (3)	1.38953 (11)	0.0519 (4)	
H6A	0.3803	−0.0985	1.3980	0.062*	
C7	0.33245 (10)	0.0997 (2)	1.30578 (11)	0.0408 (4)	
C8	0.20875 (11)	0.3377 (2)	1.00826 (11)	0.0443 (4)	
H8A	0.1736	0.4076	1.0281	0.053*	
H8B	0.2638	0.3969	1.0285	0.053*	
C9	0.16630 (10)	0.3261 (2)	0.90919 (10)	0.0385 (3)	
C10	0.15158 (10)	0.4812 (2)	0.85880 (10)	0.0393 (4)	
C11	0.11201 (10)	0.4568 (2)	0.76766 (10)	0.0405 (4)	
C12	0.08915 (12)	0.2862 (2)	0.73202 (11)	0.0493 (4)	
H12A	0.0628	0.2686	0.6715	0.059*	
C13	0.10670 (13)	0.1429 (2)	0.78931 (12)	0.0536 (5)	
H13A	0.0913	0.0278	0.7657	0.064*	
C14	0.05724 (12)	0.5917 (2)	0.62635 (11)	0.0454 (4)	
H14A	−0.0010	0.5500	0.6070	0.054*	
H14B	0.0875	0.5051	0.6076	0.054*	
C15	0.05779 (11)	0.7721 (3)	0.58855 (11)	0.0495 (4)	
C16	0.17477 (13)	0.6669 (3)	0.89758 (12)	0.0548 (5)	
H16A	0.1592	0.7539	0.8511	0.082*	
H16B	0.2352	0.6730	0.9344	0.082*	
H16C	0.1448	0.6924	0.9320	0.082*	

### Atomic displacement parameters (Å^2^)

**Table:**

	*U*^11^	*U*^22^	*U*^33^	*U*^12^	*U*^13^	*U*^23^
S1	0.0616 (3)	0.0325 (3)	0.0362 (2)	−0.00018 (16)	0.0168 (2)	0.00201 (14)
O1	0.0649 (8)	0.0411 (7)	0.0345 (6)	−0.0002 (5)	0.0143 (5)	0.0050 (5)
O2	0.0481 (7)	0.0306 (6)	0.0920 (10)	−0.0005 (5)	0.0299 (7)	0.0013 (6)
N1	0.0517 (8)	0.0324 (7)	0.0404 (7)	0.0030 (6)	0.0199 (6)	0.0036 (5)
N2	0.0523 (8)	0.0304 (7)	0.0395 (7)	0.0015 (6)	0.0172 (6)	0.0040 (5)
N3	0.0613 (9)	0.0367 (7)	0.0395 (8)	−0.0012 (6)	0.0174 (7)	0.0000 (6)
F1	0.0650 (8)	0.1046 (11)	0.1088 (12)	−0.0198 (7)	0.0357 (8)	0.0334 (9)
F2	0.0844 (8)	0.0471 (7)	0.0706 (8)	0.0098 (5)	0.0322 (7)	0.0056 (5)
F3	0.1476 (14)	0.0680 (8)	0.0367 (6)	−0.0046 (8)	0.0267 (7)	0.0095 (6)
C1	0.0419 (8)	0.0318 (8)	0.0383 (8)	0.0007 (6)	0.0172 (7)	0.0037 (6)
C1'	0.0519 (11)	0.0715 (15)	0.0859 (16)	0.0019 (10)	0.0171 (11)	0.0122 (12)
C2	0.0444 (8)	0.0411 (9)	0.0408 (9)	0.0024 (7)	0.0203 (7)	0.0022 (7)
C2'	0.0493 (10)	0.0350 (9)	0.0896 (15)	0.0018 (8)	0.0268 (10)	−0.0054 (9)
C3	0.0682 (12)	0.0485 (11)	0.0517 (11)	−0.0001 (9)	0.0280 (9)	−0.0078 (8)
C3'	0.0667 (13)	0.0789 (16)	0.0785 (15)	−0.0071 (11)	0.0369 (12)	−0.0080 (12)
C4	0.0703 (12)	0.0766 (14)	0.0434 (10)	−0.0044 (11)	0.0260 (9)	−0.0130 (9)
C5	0.0584 (11)	0.0791 (14)	0.0382 (9)	0.0024 (10)	0.0188 (8)	0.0082 (9)
C6	0.0554 (10)	0.0533 (11)	0.0442 (10)	0.0035 (8)	0.0195 (8)	0.0122 (8)
C7	0.0419 (8)	0.0412 (9)	0.0401 (8)	0.0006 (6)	0.0190 (7)	0.0044 (6)
C8	0.0566 (10)	0.0334 (8)	0.0365 (9)	0.0009 (7)	0.0147 (7)	0.0028 (7)
C9	0.0404 (8)	0.0363 (8)	0.0365 (8)	0.0010 (6)	0.0149 (6)	0.0004 (6)
C10	0.0406 (8)	0.0374 (8)	0.0367 (8)	0.0014 (6)	0.0143 (6)	0.0014 (6)
C11	0.0425 (8)	0.0388 (8)	0.0375 (8)	0.0016 (7)	0.0152 (6)	0.0044 (7)
C12	0.0595 (10)	0.0467 (10)	0.0337 (8)	−0.0012 (8)	0.0134 (7)	−0.0017 (7)
C13	0.0726 (12)	0.0376 (9)	0.0421 (9)	−0.0051 (8)	0.0176 (9)	−0.0053 (7)
C14	0.0536 (9)	0.0453 (10)	0.0341 (8)	0.0029 (7)	0.0165 (7)	0.0017 (7)
C15	0.0548 (10)	0.0539 (11)	0.0365 (8)	−0.0026 (8)	0.0170 (7)	0.0045 (7)
C16	0.0733 (12)	0.0383 (9)	0.0422 (9)	−0.0022 (8)	0.0159 (9)	0.0010 (7)

### Geometric parameters (Å, º)

**Table:**

S1—C1	1.7517 (16)	C3'—H3'A	0.9600
S1—C8	1.8088 (17)	C3'—H3'B	0.9600
O1—C11	1.366 (2)	C3'—H3'C	0.9600
O1—C14	1.414 (2)	C4—C5	1.388 (3)
O2—C2'	1.436 (2)	C4—H4A	0.9300
O2—H2B	0.8200	C5—C6	1.374 (3)
N1—C1	1.313 (2)	C5—H5A	0.9300
N1—C2	1.397 (2)	C6—C7	1.390 (2)
N2—C1	1.360 (2)	C6—H6A	0.9300
N2—C7	1.376 (2)	C8—C9	1.510 (2)
N2—H2A	0.8600	C8—H8A	0.9700
N3—C13	1.331 (2)	C8—H8B	0.9700
N3—C9	1.337 (2)	C9—C10	1.387 (2)
F1—C15	1.311 (2)	C10—C11	1.398 (2)
F2—C15	1.326 (2)	C10—C16	1.502 (2)
F3—C15	1.331 (2)	C11—C12	1.383 (3)
C1'—C2'	1.512 (3)	C12—C13	1.380 (3)
C1'—H1'A	0.9600	C12—H12A	0.9300
C1'—H1'B	0.9600	C13—H13A	0.9300
C1'—H1'C	0.9600	C14—C15	1.486 (3)
C2—C3	1.388 (3)	C14—H14A	0.9700
C2—C7	1.398 (2)	C14—H14B	0.9700
C2'—C3'	1.499 (3)	C16—H16A	0.9600
C2'—H2'A	0.9800	C16—H16B	0.9600
C3—C4	1.375 (3)	C16—H16C	0.9600
C3—H3B	0.9300		
			
C1—S1—C8	99.09 (8)	C7—C6—H6A	121.5
C11—O1—C14	117.24 (13)	N2—C7—C6	132.68 (16)
C2'—O2—H2B	109.5	N2—C7—C2	105.36 (14)
C1—N1—C2	104.42 (13)	C6—C7—C2	121.96 (17)
C1—N2—C7	106.91 (13)	C9—C8—S1	108.55 (11)
C1—N2—H2A	126.5	C9—C8—H8A	110.0
C7—N2—H2A	126.5	S1—C8—H8A	110.0
C13—N3—C9	117.35 (15)	C9—C8—H8B	110.0
N1—C1—N2	113.52 (14)	S1—C8—H8B	110.0
N1—C1—S1	128.56 (12)	H8A—C8—H8B	108.4
N2—C1—S1	117.92 (12)	N3—C9—C10	124.55 (15)
C2'—C1'—H1'A	109.5	N3—C9—C8	115.14 (14)
C2'—C1'—H1'B	109.5	C10—C9—C8	120.30 (14)
H1'A—C1'—H1'B	109.5	C9—C10—C11	116.09 (15)
C2'—C1'—H1'C	109.5	C9—C10—C16	123.33 (14)
H1'A—C1'—H1'C	109.5	C11—C10—C16	120.58 (14)
H1'B—C1'—H1'C	109.5	O1—C11—C12	123.98 (14)
C3—C2—N1	130.27 (16)	O1—C11—C10	115.48 (14)
C3—C2—C7	119.94 (16)	C12—C11—C10	120.53 (15)
N1—C2—C7	109.79 (15)	C13—C12—C11	117.74 (16)
O2—C2'—C3'	108.28 (17)	C13—C12—H12A	121.1
O2—C2'—C1'	109.59 (18)	C11—C12—H12A	121.1
C3'—C2'—C1'	112.37 (18)	N3—C13—C12	123.72 (17)
O2—C2'—H2'A	108.8	N3—C13—H13A	118.1
C3'—C2'—H2'A	108.8	C12—C13—H13A	118.1
C1'—C2'—H2'A	108.8	O1—C14—C15	107.09 (14)
C4—C3—C2	117.96 (19)	O1—C14—H14A	110.3
C4—C3—H3B	121.0	C15—C14—H14A	110.3
C2—C3—H3B	121.0	O1—C14—H14B	110.3
C2'—C3'—H3'A	109.5	C15—C14—H14B	110.3
C2'—C3'—H3'B	109.5	H14A—C14—H14B	108.6
H3'A—C3'—H3'B	109.5	F1—C15—F2	106.44 (17)
C2'—C3'—H3'C	109.5	F1—C15—F3	107.37 (16)
H3'A—C3'—H3'C	109.5	F2—C15—F3	106.68 (16)
H3'B—C3'—H3'C	109.5	F1—C15—C14	113.07 (16)
C3—C4—C5	121.61 (19)	F2—C15—C14	113.23 (14)
C3—C4—H4A	119.2	F3—C15—C14	109.68 (15)
C5—C4—H4A	119.2	C10—C16—H16A	109.5
C6—C5—C4	121.53 (18)	C10—C16—H16B	109.5
C6—C5—H5A	119.2	H16A—C16—H16B	109.5
C4—C5—H5A	119.2	C10—C16—H16C	109.5
C5—C6—C7	116.97 (19)	H16A—C16—H16C	109.5
C5—C6—H6A	121.5	H16B—C16—H16C	109.5
			
C2—N1—C1—N2	0.47 (18)	C13—N3—C9—C10	0.8 (3)
C2—N1—C1—S1	179.51 (12)	C13—N3—C9—C8	179.99 (15)
C7—N2—C1—N1	−0.99 (18)	S1—C8—C9—N3	3.65 (19)
C7—N2—C1—S1	179.85 (11)	S1—C8—C9—C10	−177.14 (12)
C8—S1—C1—N1	6.99 (17)	N3—C9—C10—C11	−0.9 (2)
C8—S1—C1—N2	−174.00 (12)	C8—C9—C10—C11	179.94 (14)
C1—N1—C2—C3	−179.33 (18)	N3—C9—C10—C16	178.21 (17)
C1—N1—C2—C7	0.23 (18)	C8—C9—C10—C16	−0.9 (2)
N1—C2—C3—C4	−178.76 (18)	C14—O1—C11—C12	0.6 (2)
C7—C2—C3—C4	1.7 (3)	C14—O1—C11—C10	−179.17 (14)
C2—C3—C4—C5	−0.3 (3)	C9—C10—C11—O1	−179.78 (13)
C3—C4—C5—C6	−1.1 (3)	C16—C10—C11—O1	1.1 (2)
C4—C5—C6—C7	1.0 (3)	C9—C10—C11—C12	0.5 (2)
C1—N2—C7—C6	−178.20 (18)	C16—C10—C11—C12	−178.69 (17)
C1—N2—C7—C2	1.05 (17)	O1—C11—C12—C13	−179.70 (17)
C5—C6—C7—N2	179.62 (17)	C10—C11—C12—C13	0.0 (3)
C5—C6—C7—C2	0.5 (3)	C9—N3—C13—C12	−0.2 (3)
C3—C2—C7—N2	178.81 (15)	C11—C12—C13—N3	−0.2 (3)
N1—C2—C7—N2	−0.81 (18)	C11—O1—C14—C15	−174.15 (14)
C3—C2—C7—C6	−1.8 (3)	O1—C14—C15—F1	59.7 (2)
N1—C2—C7—C6	178.54 (15)	O1—C14—C15—F2	−61.42 (19)
C1—S1—C8—C9	−178.94 (12)	O1—C14—C15—F3	179.55 (15)

### Hydrogen-bond geometry (Å, º)

**Table:**

*D*—H···*A*	*D*—H	H···*A*	*D*···*A*	*D*—H···*A*
O2—H2*B*···N1^i^	0.82	2.01	2.8142 (18)	167
N2—H2*A*···O2^ii^	0.86	1.98	2.8027 (18)	161

Symmetry codes: (i) *x*, *y*, *z*−1; (ii) *x*, *y*−1, *z*+1.
